# Supplementary material for: Residual force enhancement is affected more by quadriceps muscle length than stretch amplitude
Source: eLife. 2022 May 17;11:e77553. doi: 10.7554/eLife.77553 (PMC9129877; doi:10.7554/eLife.77553)
Supplement: Transparent reporting form [file elife-77553-transrepform1.docx]

***eLife’s* transparent reporting form**

We encourage authors to provide detailed information *within their submission* to facilitate the interpretation and replication of experiments. Authors can upload supporting documentation to indicate the use of appropriate reporting guidelines for health-related research (see [EQUATOR Network](http://www.equator-network.org/%20)), life science research (see the [BioSharing Information Resource](https://biosharing.org/)), or the [ARRIVE guidelines](http://www.plosbiology.org/article/info:doi/10.1371/journal.pbio.1000412) for reporting work involving animal research. Where applicable, authors should refer to any relevant reporting standards documents in this form.

If you have any questions, please consult our Journal Policies and/or contact us: [editorial@elifesciences.org](mailto:editorial@elifesciences.org).

**Sample-size estimation**

- You should state whether an appropriate sample size was computed when the study was being designed
- You should state the statistical method of sample size computation and any required assumptions
- If no explicit power analysis was used, you should describe how you decided what sample (replicate) size (number) to use

Please outline where this information can be found within the submission (e.g., sections or figure legends), or explain why this information doesn’t apply to your submission:

An a-priori sample size calculation indicated that at least thirteen participants were required to have 90% power to be able to detect a large difference (Cohen’s *d*_z_ = 1) between two dependent means with a two-tailed alpha level of 5%. This information is located at the end of the participants section within the methods.

**Replicates**

- You should report how often each experiment was performed
- You should include a definition of biological versus technical replication
- The data obtained should be provided and sufficient information should be provided to indicate the number of independent biological and/or technical replicates
- If you encountered any outliers, you should describe how these were handled
- Criteria for exclusion/inclusion of data should be clearly stated
- High-throughput sequence data should be uploaded before submission, with a private link for reviewers provided (these are available from both GEO and ArrayExpress)

Please outline where this information can be found within the submission (e.g., sections or figure legends), or explain why this information doesn’t apply to your submission:

This non-invasive *in vivo* experiment was performed on a total of sixteen human participants, who participated once. For a total of five participants, there were data collection problems with one of the experimental techniques and thus five datasets were not analyzed, which is outlined in the first paragraph of the results. Individual conditions were repeated at least three times throughout the experiment as outlined in the last paragraph of the experimental protocol. Data was excluded from statistical analysis based on the criteria outlined in the first paragraphs of the data analysis (surface EMG amplitude not matched) and statistics sections (outlier exclusion) within the methods.

**Statistical reporting**

- Statistical analysis methods should be described and justified
- Raw data should be presented in figures whenever informative to do so (typically when N per group is less than 10)
- For each experiment, you should identify the statistical tests used, exact values of N, definitions of center, methods of multiple test correction, and dispersion and precision measures (e.g., mean, median, SD, SEM, confidence intervals; and, for the major substantive results, a measure of effect size (e.g., Pearson's r, Cohen's d)
- Report exact p-values wherever possible alongside the summary statistics and 95% confidence intervals. These should be reported for all key questions and not only when the p-value is less than 0.05.

Please outline where this information can be found within the submission (e.g., sections or figure legends), or explain why this information doesn’t apply to your submission:

The statistical analysis section outlines the statistical tests we performed, which are justified based on the repeated-measures study design and distribution of paired differences following outlier removal. Data-time traces from a representative individual and individual data points are shown in the figures. The sample size for each test is outlined in the first paragraph of the results section, and the central tendency of the difference between paired groups (i.e. the mean difference) and the utilized post-hoc tests, as well as the 95% confidence interval and associated *p* value are reported. Pearson’s *r* is reported for the repeated-measures correlations along with the 95% confidence interval and *p* value.

(For large datasets, or papers with a very large number of statistical tests, you may upload a single table file with tests, Ns, etc., with reference to sections in the manuscript.)

**Group allocation**

- Indicate how samples were allocated into experimental groups (in the case of clinical studies, please specify allocation to treatment method); if randomization was used, please also state if restricted randomization was applied
- Indicate if masking was used during group allocation, data collection and/or data analysis

Please outline where this information can be found within the submission (e.g., sections or figure legends), or explain why this information doesn’t apply to your submission:

Participants were randomly selected from a recreationally-active population of young healthy students and staff that studied or worked at the Faculty of Sport Science at Ruhr University Bochum. Information about randomization can be found in the last paragraph of the experimental protocol section within the methods. Restricted randomization and masking were not applied.

**Additional data files (“source data”)**

- We encourage you to upload relevant additional data files, such as numerical data that are represented as a graph in a figure, or as a summary table
- Where provided, these should be in the most useful format, and they can be uploaded as “Source data” files linked to a main figure or table
- Include model definition files including the full list of parameters used
- Include code used for data analysis (e.g., R, MatLab)
- Avoid stating that data files are “available upon request”

Please indicate the figures or tables for which source data files have been provided:

The final processed data can be found at: <https://figshare.com/s/d66e2c7400480dd0a059>. This information is also presented in the “Data availability” statement and provides source data for figures 1, 2, 3, 4, 5, and 6.
